# Kelch proteins: emerging roles in skeletal muscle development and diseases

**DOI:** 10.1186/2044-5040-4-11

**Published:** 2014-06-01

**Authors:** Vandana A Gupta, Alan H Beggs

**Affiliations:** 1 Division of Genetics and Genomics, The Manton Center for Orphan Disease Research, Boston Children’s Hospital, Harvard Medical School, 300 Longwood Ave., Boston, MA 02115, USA

**Keywords:** Kelch, BTB, BACK, Nemaline myopathy, Dystrophy, Congenital myopathy, Cul3, Ubiquitination, Proteasome, Skeletal muscle, Proliferation, Differentiation

## Abstract

Our understanding of genes that cause skeletal muscle disease has increased tremendously over the past three decades. Advances in approaches to genetics and genomics have aided in the identification of new pathogenic mechanisms in rare genetic disorders and have opened up new avenues for therapeutic interventions by identification of new molecular pathways in muscle disease. Recent studies have identified mutations of several Kelch proteins in skeletal muscle disorders. The Kelch superfamily is one of the largest evolutionary conserved gene families. The 66 known family members all possess a Kelch-repeat containing domain and are implicated in diverse biological functions. In skeletal muscle development, several Kelch family members regulate the processes of proliferation and/or differentiation resulting in normal functioning of mature muscles. Importantly, many Kelch proteins function as substrate-specific adaptors for Cullin E3 ubiquitin ligase (Cul3), a core component of the ubiquitin-proteasome system to regulate the protein turnover. This review discusses the emerging roles of Kelch proteins in skeletal muscle function and disease.

## Review

Skeletal muscle development is a highly coordinated process that involves the myogenesis and differentiation of primary myoblasts, and their integrated growth and development into a mature functional organ [1-4]. Consequently, mutations of a large number of proteins associated with development and/or maintenance of skeletal muscle result in disease states. Over the past three decades, tremendous progress has been made in elucidating the genetic basis of muscle diseases. Primary inherited diseases of skeletal muscle include the muscular dystrophies and the non-dystrophic congenital myopathies [5-8]. Muscular dystrophies are characterized by myofiber degeneration with repeated rounds of regeneration that ultimately lead to an end-stage process typified by fibrosis and replacement by adipose tissue [9,10]. In contrast, non-dystrophic myopathies exhibit little necrotic or regenerative changes, but muscle biopsies often display characteristic structural changes such as central cores, nemaline rods, central nuclei, various intracytoplasmic inclusions, or fiber type disproportion, and so on [10,11]. Collectively, these diseases are both phenotypically and clinically heterogeneous. Gene discovery in muscle diseases is currently skyrocketing due to the use of next-generation sequencing approaches [12-19]. The discovery of new genes is not only crucial for improving diagnostics for these highly heterogeneous muscular disorders, but also is critical for identifying new molecular pathways that may serve as potential therapeutic targets. Recent gene discoveries have identified mutations in Kelch protein genes as the cause of muscle diseases in humans [14,20-22]. Kelch proteins belong to the Kelch superfamily that consists of a large number of structurally and functionally diverse proteins characterized by the presence of a Kelch-repeat domain [23,24]. Kelch family members are involved in a number of cellular and molecular processes such as cell migration, cytoskeletal arrangement, regulation of cell morphology, protein degradation, and gene expression [25-31]. This review summarizes our emerging understanding of the various roles of Kelch proteins in skeletal muscle development and disease (Tables 1 and 2).

**Table 1:** Kelch family proteins in skeletal muscle development

**Gene**	**Protein**	**Function**	**Expression**
***KLHL19***	KLHL19, KEAP1	Oxidative stress and insulin signaling in muscle cells [32]	Ubiquitous [33]
***KLHL31***	KLHL31	Skeletal and cardiac muscle myogenesis [29,34]	Skeletal muscle, heart (low levels in brain, kidney, and liver) [29]
***KLHL39***	KLHL39, IVNS1ABP	Protection against drug-induced cardiomyopathy [35]	Ubiquitous [36]
***KLHL40 (KBTBD5)***	KLHL40	Skeletal muscle differentiation [14,37]	Skeletal muscle [14]
***KLHL41 (KBTBD10****,* ***KRP1)***	KLHL41, Sarcosin	Skeletal muscle differentiation and myofibril assembly [22,38,39]	Skeletal muscle, lungs [22]
***MKLN1***	MKLN1, Muskelin	Muscle cell adhesion and extracellular communication [40]	Skeletal muscle, brain [40,41]
***KLHDC1***	KLHDC1	Muscle cell migration and differentiation [42,43]	Skeletal muscle [42]
***KLHDC2***	KLHDC2	Muscle cell migration and differentiation [42,43]	Skeletal muscle [42]

**Table 2:** Kelch family proteins in human diseases

**Gene**	**Function**	**Expression**
***Neuromuscular diseases***		
***KLHL1***	Spinocerebellar ataxia type 8 [44]	Brain, prostate, small intestine, colon [44]
***KLHL9***	Distal myopathy [20]	Ubiquitous [20]
***KLHL16 (GAN)***	Giant axonal neuropathy [45]	Brain, skeletal muscle, heart, kidney, liver [46]
***KLHL40 (KBTBD5)***	Severe nemaline myopathy with fetal akinesia [14,37]	Skeletal muscle [14]
***KLHL41 (KBTBD10)***	Nemaline myopathy [22,38,39]	Skeletal muscle, lungs [22]
***KBTBD13***	Nemaline myopathy with cores [21]	Skeletal muscle, lungs, heart [21]
***Cancer***		
***KLHL6***	Chronic lymphocytic leukemia [47]	Lymphocytes (unknown in other tissues) [48]
***KLHL19 (KEAP1)***	Pulmonary papillary adenocarcinoma [49]	Ubiquitous [50]
***KLHL20***	Prostate cancer progression [51]	Ubiquitous [52]
***KLHL37 (ENC1)***	Brain tumors [53]	Brain (unknown in other tissues) [54]
***KLHDC8B***	Hodgkin’s lymphoma [55]	Unknown
***Other Inherited Diseases***		
***KLHL3***	Pseudohypoalsosteronism type II [56]	Cerebellum, kidney, spinal cord, heart, lung, placenta, testis, arota [56]
***KLHL7***	Autosomal dominant retinitis pigmentosa [57]	Ubiquitous [57]
***KLHL10***	Oligozoospermia [58,59]	Testis [60]

### Kelch protein superfamily

**Figure 1:**
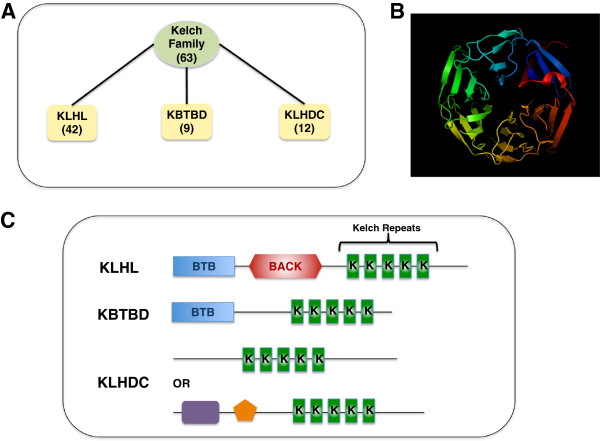
**The Kelch Superfamily. (A)** The Kelch family consists of 63 proteins that are subclassified in to KLHL, KBTBD and KLHDC subfamilies. **(B)** Structure of Kelch domain of rat KLHL41 (PDB code 2WOZ) comprising six repeats that form the complete Kelch domain. The structure was generated using PyMOL (http://www.pymol.org). **(C)** Prototype members of different subfamilies showing different domain organization. KLHL proteins have an N-terminal BTB/POZ, a BACK and C-terminal Kelch repeats. KBTBD proteins contain an N-terminal BTB domain and Kelch repeats. The BACK domain is normally absent in KBTBD proteins. KLHDC proteins lack both BTB/POZ and BACK domains and contain either Kelch repeats alone or with other domains such as transmembrane (for example, KLHDC7A), Glycine rich (for example, KLHDC10), or Lish and CTLH domains (for example, MKLN1).

**Figure 2:**
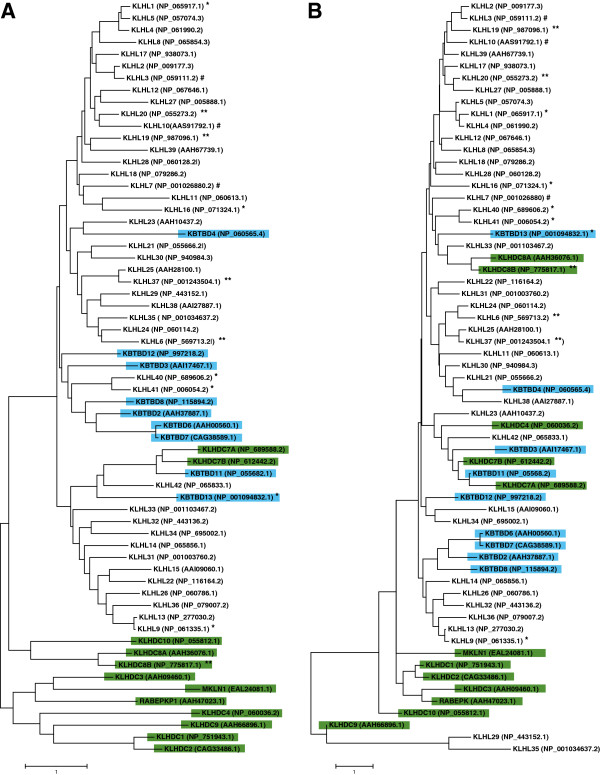
**Phylogenetic analysis showing relationships between human Kelch protein family members. (A)** Phylogenetic tree of full-length amino acid sequences of human proteins were aligned. **(B)** Phylogenetic tree of amino acid sequences of Kelch domains. Phylogenetic trees were constructed by maximum-likelihood method using BLOSUM matrix in MEGA 6.06. Reference sequences used for alignments are indicated at right of each protein name. Blue highlighting indicates KBTBD subfamily members; green indicates KLHDC subfamily members. *, proteins involved in neuromuscular diseases; **, family members implicated in cancer; #, proteins whose defects cause other inherited diseases (Table 2). Scale bars indicate relative distances and represent the degree of differences between the sequences.

The Kelch superfamily includes 66 genes, of which 63 are protein-coding whereas three are non-coding genes. The Kelch protein family is primarily classified into KLHL, KBTBD, and KLHDC subfamilies that differ in the types and numbers of their protein domains (http://www.genenames.org/) (Figure 1A). A prototypic member of the Kelch family contains an N-terminal BTB/POZ domain, a BACK domain, and two to eight C-terminal Kelch motifs [61]. KLHL subfamily members contain all of these domains whereas the KBTBD subfamily members typically lack the BACK domain [24]. KLHDC subfamily members do not contain either BTB or BACK domains (Figure 1B and 1C). However, some KLHDC subfamily members do contain alternative domains, such as the transmembrane (KLHDC7A), Glycine rich (KLHDC10), Lish and CTLH (MKLN1) domains, in addition to traditional Kelch repeats.A phylogenetic analysis of human Kelch family proteins shows that most members within each subfamily largely cluster together with the KLHDC subfamily, that lacks BTB and BACK domains, apparently having diverged first (Figure 2A). However, several members are clustered with other subfamilies suggesting that some gain or loss of BTB or BACK domains may have occasionally occurred at later stages of evolution. Kelch proteins bind to specific substrates through their Kelch domains. An amino acid alignment of only the Kelch repeat regions shows that proteins involved in similar disease processes are clustered together (for example, KLHL40, KLHL41, and KBTBD13, all of which cause nemaline myopathy) (Figure 2B). This suggests that Kelch domains with high sequence homology may be regulating similar biological processes through interactions with shared or related binding partners. Under this model, functional differences between these related Kelch family members will likely derive from variation in other domains of the proteins.

#### BTB/POZ domain

The BTB/POZ domain derives its name from the Drosophila Bric-a-brac, Tramtrac, and Broad complex due to sequence homology [62,63]. As the DNA poxvirus in which this domain was first identified also showed some similarity to zinc finger proteins, this domain was concurrently named the POZ (Poxvirus and Zinc finger) domain. BTB domains are mainly involved in facilitating self-oligomerization or mediating protein-protein interactions with other proteins [64,65]. Despite the similar secondary structures and shared organization of BTB proteins, their primary sequences are not well conserved. This sequence variability between BTB proteins contributes to differential protein-protein interactions and results in different functional roles. BTB domains are also present in other non Kelch-repeat containing proteins. The major difference lies in the protein interactors of BTB domains in Kelch *versus* non-Kelch proteins. In the Kelch family, the most important known interactive role of the BTB domain is to act as an adaptor between E3 ubiquitin ligases and Kelch domains in order to form active ubiquitination complexes [66-68]. In non-Kelch families of proteins, BTB protein-regulated interactions typically involve recruitment of co-activators or repressors to transcription complexes. In addition, these interacting proteins are also involved in cytoskeletal arrangement and ion conductance [69-72].

#### BACK domain

The BACK domains found in KLHL subfamily members are the most conserved domain of the Kelch family and are present between the N-terminal BTB and C-terminal Kelch domains. BACK domains contain an N-terminal conserved Asn-Cys-Leu-Gly-Ile motif and a Val-Arg-[Leu/Met/Phe]-Pro-Leu-Leu sequence, two arginines, four glutamic acids, and several hydrophobic positions causing them to be hydrophobic in nature [61]. The true role of the BACK domain is not known, but it is predicted to participate in BTB-E3 ubiquitin ligase complex formation [61]. Evidence for the functional significance of BACK domains comes from recent studies where missense mutations in this domain in KLHL40 and KLHL41 are pathogenic in human patients affected with nemaline myopathy [14,22].

#### Kelch domain

Kelch motifs range from 44 to 56 amino acids in length and are usually arranged in a series of five to seven repeats in most of the family members [23]. The signature motifs in each Kelch repeat are a series of four hydrophobic amino acids followed by glycine doublet, a conserved tyrosine, and a conserved tryptophan. Each Kelch repeat folds into four twisted antiparallel β-strands connected by intrablade loops to form a single blade of a β-propeller (Figure 1B). A C-terminal strand closure mechanism links the first and last blades to complete the propeller. Kelch β-propellers primarily function as scaffolds for protein-protein interactions. Despite the shared tertiary structure, there is little primary sequence identity between one Kelch repeat and another, suggesting a wide diversity of interacting partners across the Kelch superfamily. In nemaline myopathy, all pathogenic *KBTBD13* mutations identified to date in human patients were found in the Kelch repeats [21]. Similarly, mutations in Kelch repeats of both *KLHL40* or *KLHL41* also result in nemaline myopathy [14,22].

### Kelch proteins in skeletal muscle diseases

#### KLHL9

The first Kelch protein defect reported in a primary skeletal muscle disease was of KLHL9, resulting in an early onset autosomal dominant form of distal myopathy [20]. Distal myopathies are a heterogeneous group of muscle diseases characterized by progressive muscular atrophy and weakness, beginning in distal (hence the name) and progressing to proximal limb muscles [73]. By linkage analysis and subsequence candidate sequencing, Cirak *et al.* identified a p.Leu95Phe missense change in the N-Terminal BTB domain of KLHL9 in several affected members of a single large German family with a dominant form of distal myopathy [20]. Leucine is conserved at this position in other vertebrates, and molecular modeling predicted that the change to phenylalanine would affect BTB domain conformation and likely disrupt the protein-protein interactions with Cul3 ubiquitin ligase. Cell culture studies subsequently showed that wild-type KLHL9 interacted with Cul3 ubiquitin ligase whereas the p.Leu95Phe mutation disrupted the KLHL9-Cul3 interaction [20,74]. The Kelch family members act as substrate-recognizing adaptors of the Cul3 ubiquitination complex, which targets specific substrates for ubiquitination and subsequent degradation by proteasomal pathways. While experimental evidence for KLHL9 as a regulator of ubiquitination in skeletal muscles is still lacking, one potential substrate for KLHL9 action is postulated to be Aurora B kinase [20]. Previous studies have shown that KLHL9 targets this kinase during mitotic progression and cytokinesis [75]. Aurora B kinase is a regulator of assembly and disassembly of type III intermediate filaments including vimentin, desmin, and type IV neurofilaments. Therefore, a mis-regulation of Aurora B Kinase function in KLHL9 deficiency is postulated to affect normal skeletal muscle function [76].

#### KBTBD13

KBTBD13 is a member of the KBTBD subfamily of Kelch proteins that contains a BTB domain and Kelch repeats but lacks a BACK domain. *KBTBD13* mutations result in an autosomal dominant form of nemaline myopathy associated with cores in affected patients [21]. Clinically, patients with *KBTBD13* mutations exhibit poor exercise tolerance, gait abnormality, and progressive weakness of the neck and proximal limb muscles. The peculiar clinical feature specific to *KBTBD13* patients that is not seen in other forms of congenital myopathies is a characteristic slowness of movement, where patients are unable to run and correct themselves from falling over. Moreover, skeletal muscles of patients with *KBTBD13* mutations exhibit cores that lack oxidative enzymatic activity and show predominance as well as hypertrophy of type 1 (slow) myofibers [21]. The localization of KBTBD13 in myofibers is not clear. Localization studies have shown that KBTBD13 does not co-localize with α-actin (thin filament protein) or α-actinin (a Z-line marker), suggesting a different localization and mechanism of action than previously known proteins in nemaline myopathy, which primarily localize to thin filaments [21]. All known pathogenic mutations in *KBTBD13* are localized in the Kelch repeats of the protein and are predicted to damage the β-propeller blades. KBTBD13 forms a complex with Cul3 ubiquitin ligase through its N-terminal BTB domain and this interaction is required for the formation of a functional Cul3 ubiquitin ligase complex, suggesting that the pathogenic mechanism in KBTBD13-related nemaline myopathy may involve dysregulation of cellular protein ubiquitination [66].

#### KLHL40

*KLHL40* mutations have been recently identified as a cause of nemaline myopathy in a severe form of the disease [14]. KLHL40 belongs to the KLHL subfamily of Kelch proteins that contain an N-terminal BTB domain, a BACK domain, and five Kelch repeats on the C-terminal end. Recessive mutations in *KLHL40* are associated with fetal akinesia or hypokinesia during the prenatal period, respiratory failure, and swallowing difficulty at birth, as well as contractures, fractures, and dysmorphic features that in many cases are associated with early death. A significant number of patients with *KLHL40* mutations (approximately 17%) also exhibit ophthalmoparesis that is usually not present in other genetic subtypes of nemaline myopathy. Moreover, fractures are also a relatively frequent presentation in *KLHL40* patients that is not commonly seen in other forms of nemaline and other congenital myopathies. Mutations of *KLHL40* have been found in exons encoding all domains of the protein with a majority of the mutations seen in the Kelch repeats, including a founder mutation (p.Glu528Lys) present in Japanese, Turkish, and Kurdish populations. Both truncating as well as missense mutations are seen in *KLHL40*. Knockdown of *KLHL40* orthologues in zebrafish results in sarcomeric abnormalities and impaired locomotion similar to human patients, providing further evidence that *KLHL40* is the disease-causing gene in nemaline myopathy [14]. Murine Klhl40 protein is localized at triads along with the sarcoplasmic reticulum (SR) marker Ryr1 [22]. KLHL40 interacts with CUL3 ubiquitin ligase via the N-terminal BTB domain and forms a protein complex, the functional significance of which remains to be unraveled [68]. Recent work has also shown that *Klhl40* promoter is a direct target of myoD and is crucial for muscle cell differentiation [22,37].

#### KLHL41

KLHL41 is the most recent member of the Kelch family to be implicated in muscle disease. Unlike *KLHL9* and *KBTBD13* mutations that cause a dominant form of the disease, mutations in *KLHL41*, like *KLHL40*, result in an autosomal recessive form of nemaline myopathy. Genetic analysis of five unrelated families has shown that *KLHL41* mutations appear to follow a genotype-phenotype correlation. Mutations that led to truncated proteins resulted in a severe form of the disease with fetal akinesia, a lack of antigravity movement, arthrogryposis, and dislocation of the hip and knees. These patients died within the first few months of life due to respiratory insufficiency. Missense changes resulted in a mild or intermediate form of the disease with impaired motor functions and survival into late childhood and/or early adulthood. KLHL41 localizes in the perinuclear area and over (but not within) I bands, in association with the terminal cisternae and longitudinal vesicles of the SR membranes present in the I-band area at the triadic regions. As most of the previously known NM proteins are components of sarcomeric thin filaments, the unique localization of KLHL41, as well as non-sarcomeric localization of KLHL40 in association with the triads, suggests the involvement of new pathophysiological mechanisms for nemaline myopathy [77]. Knockdown of *KLHL41* in zebrafish resulted in skeletal muscle myopathy with disorganized and thinner myofibers as well as reduced motor function in comparison to wild-type zebrafish. Interestingly, morphant fish also exhibited numerous electron dense structures in skeletal muscle, reminiscent of small or nascent nemaline bodies, in addition to Z-line thickening as seen in human nemaline myopathy patients.

Previous studies have shown that KLHL41 interacts with Cul3 ubiquitin ligase to form functional ubiquitination complexes with proteins targeted for degradation [78]. The identification of protein substrates targeted by the KLHL41-Cul3 ubiquitin complex is not known. KLHL41 interacts with nebulin [79] and co-localizes with actin [80], mutations of which cause approximately 65% to 70% of all known mutations in patients affected with nemaline myopathy [81]. The high degree of sequence similarity between Kelch repeats of KBTBD13, KLHL40, and KLHL41 suggests that they may share identical or closely related binding partners whose dysregulation leads to nemaline myopathy through a common final pathway and implicates a critical role for BTB-Kelch family members in the maintenance of sarcomeric integrity in skeletal muscle.

### Kelch proteins in skeletal muscle development

Members of the Kelch family are known to be involved in multiple biological processes such as migration, cytoskeletal arrangement, regulation of cell morphology, myofibril assembly, protein degradation, and gene expression [25,27,45,80,82,83]. Several Kelch proteins associate with the actin cytoskeleton via the β-propeller module, and these associations are important for functional roles of these proteins [84,85]. Other Kelch proteins affect the organization of cytoskeletal, plasma membrane, or organelle structures but do not bind directly to or co-localize with actin.

In skeletal muscle, many Kelch proteins are known to regulate the proliferation as well as differentiation of muscle cells. KLHL41 is highly expressed in myoblasts during early muscle differentiation [86]. Knockdown as well as overexpression of *KLHL41* in C2C12 cells inhibited myoblast differentiation, suggesting a role in cell cycle exit and the promotion of differentiation [38]. Knockdown of *KLHL41* in cultured cardiomyocytes affected lateral fusion of myofibrils resulting in thin myofibrils [39]. In developing chicken and zebrafish embryos, *KLHL31* is specifically expressed in early heart and in developing myoblasts shortly after their commitment to this fate, signifying an important role during skeletal muscle and cardiac myogenesis [29]. The expression of *KLHL31* is initiated just after MyoD in developing skeletal muscles and this expression persisted in later stages of development implying the possible involvement of *KLHL31* in the early phases of myogenic commitment and during later muscle differentiation [34]. KLHL31 acts as a transcriptional repressor in the MAPK/JNK signaling pathway in mouse cardiomyocytes. In skeletal muscle cells, overexpression of KLHDC2 rendered these cells unable to respond to chemoattractants and led to augmented stress fiber formation and cell adherence [26]. Additionally, myoblasts overexpressing KLHDC2 failed to differentiate into mature myotubes. KLHDC2 also regulates transcription processes by inducing expression of the leucine zipper transcription factor (LZIP) in muscle cells. KLHDC1 is a paralogue of KLHDC2 and shares 50% similarity with KLHDC2. Similar to KLHDC2, the highest expression of KLHDC1 is observed in skeletal muscles [42]. However, the functional roles of KLHDC1 remain unknown. Muskelin (*MKLN1*, a KLHDC subfamily member) was identified in an expression cloning screen for molecules that promoted cell adhesion to the extracellular matrix component thrombospondin 1. Overexpression or antisense depletion of muskelin in mouse skeletal myoblasts correlates with altered organization of fascin microspike-based adhesive contacts and a redistribution of focal contact in TSP-1 adherent cells, yet has modest effects on actin organization in cells adherent on fibronectin [40]. These results suggest that muskelin functions at a node point in the integration of cell responses to complex extracellular matrix. As most of muskelin is cytoplasmic, its effects on matrix contacts are thought to be mediated indirectly. The requirement of Kelch proteins in cell morphology comes from yeast-based studies where mutations in Kelch proteins resulted in cell fusion defects [87]. The emerging roles of Kelch proteins in metabolic pathways come from a recent study where *Keap1* (*Klhl19*) knockout mice exhibited a significant reduction in insulin signaling pathway gene expression in skeletal muscles [32,88,89]. Keap1 also regulates redox signaling, as increased oxidative stress thought to be associated with its dysregulation is observed in sedentary elderly people [89]. Nd1-L (*KLHL39*) is an actin binding protein expressed in high levels in heart that protects against doxorubicin-induced cardiomyopathy in mice [35].

### Molecular pathways regulated by Kelch proteins

Detailed studies on molecular functions of Kelch proteins are still largely lacking except with regard to their roles as substrate specific adaptors in the ubiquitination pathways, thereby regulating diverse cellular processes [21,28,90-94]. Skeletal muscle proteins are especially prone to wear and tear as they are the primary force generating mechanism in skeletal muscle. A quality control check, by removal of damaged proteins and coordinated turnover, is performed by many proteolytic systems in skeletal muscles: the caspase and calpain systems of partially muscle-specific proteases; the ubiquitin proteasome system (UPS) which degrades polyubiquitinated proteins via the 26S proteasome; and the autophaghy pathway which removes proteins by inclusion in autophagic/lysosomal vesicles [95-104]. Kelch proteins function by acting as substrate specific adaptors for the Cullin3-dependent E3 ubiquitination ligase complex during protein ubiquitination [93,105,106].

Ubiquitination is a multistep process in which an E1 ubiquitination activating enzyme transfers ubiquitin to an E2 ubiquitin-conjugating enzyme. The final step of this process is the ligation of ubiquitin to the substrate, catalyzed by an E3 ubiquitin protein ligase [107-109]. As the stability of a large number of proteins is controlled by the ubiquitin system, it is crucial to determine how the cell achieves sufficient diversity among E3s so that each one selectively recognizes only one or a few substrates in the sea of cellular proteins present at any time. Several hundreds of such E3 ligases are described, some of which are clearly specific to skeletal muscles [110,111]. One subgroup of E3 ligases are the ubiquitously expressed cullins that do not bind to their substrates directly, but rely on an array of adaptor proteins. There are seven cullin genes known in mammals (*CUL1*-*CUL3*, *CUL4a*, *CUL4b*, *CUL5*, and *CUL7*), but only CUL3 interacts with the BTB-Kelch proteins [112]. Thus, BTB-domain proteins serve as the substrate specific adaptors of CUL3-based E3 ubiquitin ligases [113]. The crucial requirement of CUL3 function is evident from *Cul3* null mice that exhibit embryonic lethality. In particular, *Cul3* null homozygous mice exhibit abnormal cycling of cells in extraembryonic membranes, reduced size, abnormal gastrulation and trophoblast cells, absence of an amnion, and death by embryonic day 7.5 [114]. In humans, dominant CUL3 mutations are associated with pseudohypoaldosteronism type II, a genetic disorder causing hypertension, hyperkalemia, and metabolic acidosis [92]. Even though CUL3 is ubiquitously expressed, the functional specificity of CUL3 in different spatial and developmental contexts is exhibited through protein-protein interaction with different Kelch proteins. This may in part be regulated by tissue specific expression, and substrate specificity of the different Kelch protein-binding partners. KLHL40 and KLHL41 are primarily expressed in skeletal (with low expression in cardiac) muscles. In skeletal muscles, deficiency of these proteins and thereby a perturbation of Cul3 ubiquitin complex formation to regulate the protein turnover of specific substrates may lead to skeletal muscle diseases.

**Figure 3:**
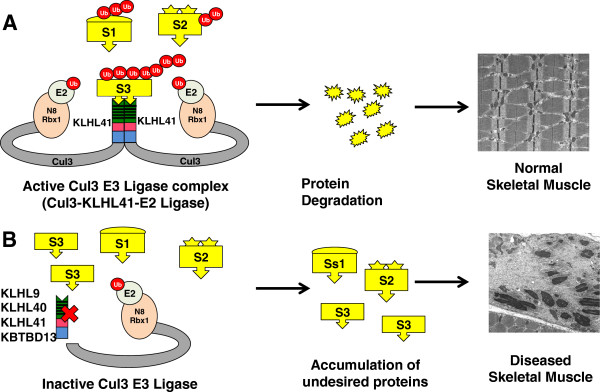
**Kelch proteins act as a substrate specific adaptors for E3-ubiquitinin protein complex. (A)** Cullin3 complex is Nedd8 (N8) modified and recruits E2-bound ubiquitin through RING-finger protein Rbx1. The assembly of a functional ubiquitination complex requires the binding of Cul3-E2 complex to substrate specific Kelch adaptor proteins. Cul3 directly binds to N-terminal BTB domain of Kelch protein and this E3-ubiquitination complex interacts with substrates (for example, S1, S2, S3) by C-terminal Kelch-repeat containing domains of Kelch proteins, causing ubiquitination of the target proteins and subsequent degradation (or stabilization and so on) by the proteasome system. This results in normal protein turnover of proteins required for normal functioning of muscle resulting in healthy skeletal muscles. **(B)** The deficiency of Kelch proteins (such as disease causing KLHL9, KLHL40, KLHL41, and KBTBD13) prevents the assembly of functional Cullin3 ubiquitination complex thereby perturbing the protein turnover process. In the model shown here, this results in accumulation of abnormal proteins (for example, S1, S2, S3) leading to unavailability of normal proteins in skeletal muscle leading to a diseased state.

The regulation of ubiquitination by Kelch proteins in skeletal muscles has been investigated in the context of disease. Kelch proteins such as KLHL9, KBTBD13, KLHL40, and KLHL41 that cause skeletal muscle disorders, each form protein complexes with CUL3 [20,66,68,78]. The functional significance of these interactions has been shown by several studies, as Cul3-KLHL41 and Cul3-KBTBD13 complexes are able to perform *in vitro* ubiquitination in the presence of other members of the functional ubiquitination complex [66,78]. Moreover, in distal myopathy, the interaction of mutant KLHL9 with Cul3 is highly reduced in comparison to wild-type KLHL9, which may in turn affect the ubiquitination of cellular proteins [20]. Identifying the protein interactors of each of these Kelch proteins in skeletal muscle will provide further insight into disease mechanisms. A study of the skeletal muscle interactome showed that KLHL41 interacts with TCAP, ENO3, and SGCG, three proteins encoded by genes mutated in limb-girdle muscular dystrophy type 2G, metabolic myopathy, and limb-girdle muscular dystrophy type 2C, respectively [79]. This points toward the potential regulatory roles of Kelch proteins and/or ubiquitination pathways in other muscle diseases as well [79,115-117]. KLHL41 also interacts with nebulin, a known causative gene of nemaline myopathy, by protein-protein interactions and is co-localized with actin at the tips of pseudopodia in fibroblasts. These data suggest that KLHL41 and other disease-causing Kelch proteins KLHL40, KBTBD13, and KLHL9 may contribute to disease pathogenesis by regulating the protein turnover of nebulin, actin, and other important skeletal muscle proteins that are required for normal functioning of skeletal muscle (Figure 3). In the absence of Kelch proteins, disturbance of this protein turnover process and subsequent overabundance of damaged sarcomeric proteins may result in disease states in affected muscles (Figure 3). In addition to protein degradation, ubiquitination has non-degradative roles such as modulation of protein activity, interaction, and sub-cellular localization of proteins [118,119], so it may be that some of these Kelch proteins actually promote the stability of their binding partners. Future studies will help us to better understand these processes in the context of skeletal muscle development and diseases.

### Kelch proteins in other human diseases

A number of Kelch proteins play important roles in a variety of human diseases, including cancer and neurological disorders [55,120-122] (Table 2). Mutations of *KLHL6*, *KLHL19*, *KLHL20*, and *KLHL37* are associated with various forms of cancers [47,49,51,53]. Whole exome sequencing in a rare genetic disorder, pseudohypoalsosteronism type II, involving hypertension, hyperkalemia, and metabolic acidosis, recently identified mutations in the *KLHL3* gene [56,92]. *KLHL3* mutations are either recessive or dominant in nature. The recessive mutations are distributed throughout the encoded protein, whereas dominant mutations were localized in the cullin binding sites, likely impairing the formation of active Cul3 complexes. Linkage analysis and mutation screening in 502 retinopathy probands identified *KLHL7* as a cause of autosomal dominant retinitis pigmentosa [57]. Interestingly, the missense mutations localized in the BACK domain of the KLHL7 protein were predicted to affect the ubiquitination complexes [57]. *KLHL10* is specifically expressed in testis and mutations of this gene lead to oligozoospermia and male infertility. Missense changes in KLHL10 impair homodimerization that is required for normal protein function, resulting in functional deficiency in patients [58]. Spinocerebellar ataxia type 8 (SCA8) is a dominantly inherited disorder caused by large CTG repeat expansions in the untranslated antisense RNA of the *KLHL1* gene [123]. While the molecular disease mechanism is still unclear, reduction of KLHL1 in mice leads to the degeneration of Purkinje cell function, suggesting a significant role in the pathophysiology of SCA8 [124]. Giant axonal neuropathy (GAN) is a rare autosomal recessive progressive neurodegenerative disorder involving the peripheral and central nervous systems. A number of mutations in gigaxonin (*GAN*), a BTB-Kelch protein, have been identified as resulting in a generalized disorganization of cytoskeletal intermediate filaments [45,125]. This cytoskeletal disorganization is attributed to an accumulation of abnormal proteins that are normally degraded by ubiquitinin-proteasome pathway in gigaxonin deficiency [126]. Collectively, these observations suggest that similar molecular pathways may be involved in the pathophysiology of a variety of diseases caused by abnormalities of Kelch proteins.

## Conclusions

Recent developments in gene discovery have led to the identification of Kelch family members as regulators of skeletal muscle development and function. We are still in the early stages of fully understanding the molecular pathways regulated by these proteins. Nonetheless, the discovery of these proteins in skeletal muscle diseases and function is very exciting, both in terms of improving diagnostics and opening up new directions of research on cellular and molecular pathways crucial for skeletal muscle function. Further studies on ubiquitination and the identification of Kelch substrates in skeletal muscle will help us to develop suitable therapeutics for a wide range of muscle diseases and related disorders.

## Competing interests

The authors declare that they have no competing interests.

## Authors’ contribution

VAG and AHB wrote the manuscript. Both authors read and approved the final manuscript.

## Authors’ information

Alan H Beggs, PhD is Sir Edwin and Lady Manton Professor of Pediatrics at Harvard Medical School and Research Associate in the Division of Genetics and Genomics at Boston Children’s Hospital where he is also director of The Manton Center for Orphan Disease Research. Dr. Beggs’ research is focused on developing approaches to diagnose and treat congenital myopathies and related neuromuscular diseases. Dr. Beggs has been associated with the discovery of several new genes associated with congenital muscle diseases as well as developing gene and protein based therapies for X-linked myotubular myopathy.

Vandana A Gupta, PhD is an Instructor in Pediatrics at Harvard Medical School and Associate Research Staff in the Division of Genetics and Genomics at Boston Children’s Hospital. Dr. Gupta has identified several new genes in congenital muscle diseases using zebrafish and human studies. Dr. Gupta has been awarded many fellowships and conference awards and is currently supported by a K01 AR062601 from the National Institute of Arthritis and Musculoskeletal and Skin Diseases of National Institutes of Health and Charles H. Hood Child Health Grant Foundation.
